# Pro-Brain-Derived Neurotrophic Factor (BDNF), but Not Mature BDNF, Is Expressed in Human Skeletal Muscle: Implications for Exercise-Induced Neuroplasticity

**DOI:** 10.1093/function/zqae005

**Published:** 2024-01-27

**Authors:** Sebastian Edman, Oscar Horwath, Thibaux Van der Stede, Sarah Joan Blackwood, Isabel Moberg, Henrik Strömlind, Fabian Nordström, Maria Ekblom, Abram Katz, William Apró, Marcus Moberg

**Affiliations:** Åstrand Laboratory, Department of Physiology, Nutrition and Biomechanics, Swedish School of Sport and Health Sciences, Stockholm 114 33, Sweden; Department of Women’s and Children’s Health, Karolinska Institute, Stockholm 171 77, Sweden; Åstrand Laboratory, Department of Physiology, Nutrition and Biomechanics, Swedish School of Sport and Health Sciences, Stockholm 114 33, Sweden; Department of Movement and Sport Sciences, Ghent University, Ghent 9000, Belgium; The August Krogh Section for Human Physiology, Department of Nutrition, Exercise and Sports, University of Copenhagen, Copenhagen 1172, Denmark; Åstrand Laboratory, Department of Physiology, Nutrition and Biomechanics, Swedish School of Sport and Health Sciences, Stockholm 114 33, Sweden; Åstrand Laboratory, Department of Physiology, Nutrition and Biomechanics, Swedish School of Sport and Health Sciences, Stockholm 114 33, Sweden; Åstrand Laboratory, Department of Physiology, Nutrition and Biomechanics, Swedish School of Sport and Health Sciences, Stockholm 114 33, Sweden; Åstrand Laboratory, Department of Physiology, Nutrition and Biomechanics, Swedish School of Sport and Health Sciences, Stockholm 114 33, Sweden; Department of Physical Activity and Health, Swedish School of Sport and Health Sciences, Stockholm 114 33, Sweden; Department of Neuroscience, Karolinska Institute, Stockholm 171 77, Sweden; Åstrand Laboratory, Department of Physiology, Nutrition and Biomechanics, Swedish School of Sport and Health Sciences, Stockholm 114 33, Sweden; Åstrand Laboratory, Department of Physiology, Nutrition and Biomechanics, Swedish School of Sport and Health Sciences, Stockholm 114 33, Sweden; Department of Clinical Science, Intervention and Technology, Karolinska Institute, Stockholm 171 77, Sweden; Åstrand Laboratory, Department of Physiology, Nutrition and Biomechanics, Swedish School of Sport and Health Sciences, Stockholm 114 33, Sweden; Department of Physiology and Pharmacology, Karolinska Institute, Stockholm 171 77, Sweden

**Keywords:** neurotrophins, exercise, muscle fiber type, lactate, β-hydroxybutyrate, fasting

## Abstract

Exercise promotes brain plasticity partly by stimulating increases in mature brain-derived neurotrophic factor (mBDNF), but the role of the pro-BDNF isoform in the regulation of BDNF metabolism in humans is unknown. We quantified the expression of pro-BDNF and mBDNF in human skeletal muscle and plasma at rest, after acute exercise (+/− lactate infusion), and after fasting. Pro-BDNF and mBDNF were analyzed with immunoblotting, enzyme-linked immunosorbent assay, immunohistochemistry, and quantitative polymerase chain reaction. Pro-BDNF was consistently and clearly detected in skeletal muscle (40-250 pg mg^−1^ dry muscle), whereas mBDNF was not. All methods showed a 4-fold greater pro-BDNF expression in type I muscle fibers compared to type II fibers. Exercise resulted in elevated plasma levels of mBDNF (55%) and pro-BDNF (20%), as well as muscle levels of pro-BDNF (∼10%, all *P* < 0.05). Lactate infusion during exercise induced a significantly greater increase in plasma mBDNF (115%, *P* < 0.05) compared to control (saline infusion), with no effect on pro-BDNF levels in plasma or muscle. A 3-day fast resulted in a small increase in plasma pro-BDNF (∼10%, *P* < 0.05), with no effect on mBDNF. Pro-BDNF is highly expressed in human skeletal muscle, particularly in type I fibers, and is increased after exercise. While exercising with higher lactate augmented levels of plasma mBDNF, exercise-mediated increases in circulating mBDNF likely derive partly from release and cleavage of pro-BDNF from skeletal muscle, and partly from neural and other tissues. These findings have implications for preclinical and clinical work related to a wide range of neurological disorders such as Alzheimer’s, clinical depression, and amyotrophic lateral sclerosis.

## Graphical Abstract

**Graphical Abstract:**
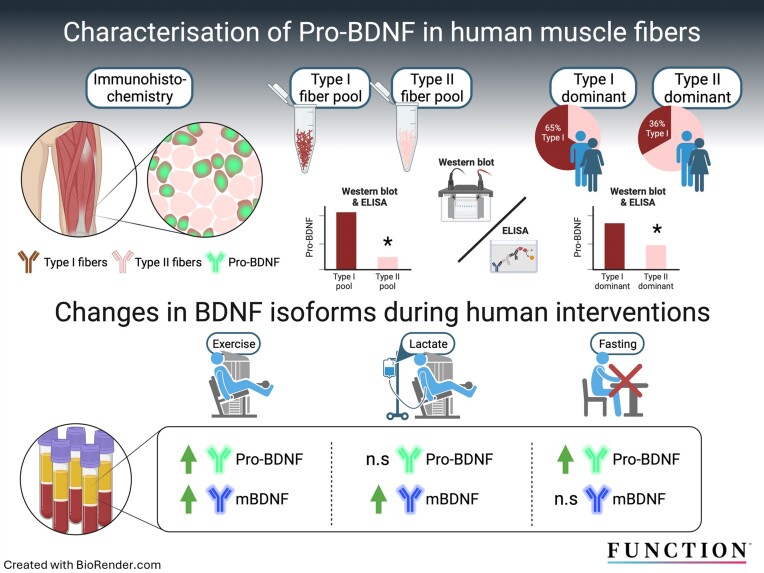

## Introduction

Brain-derived neurotrophic factor (BDNF) belongs to the family of neurotrophins and stimulates neuronal growth and differentiation, synaptogenesis, and neural plasticity.^1^ Brain-derived neurotrophic factor is synthesized as an isoform termed pro-BDNF, which is reported to have a molecular mass of 28-32 kDa, where the canonical sequence is 28 kDa.^2,3^ Pro-BDNF can be cleaved, both intra- and extracellularly, to the 14-kDa mature BDNF (mBDNF or conventionally just BDNF) and a 17-kDa pro-peptide.^3^ Pro-BDNF is biologically active and binds to the p75^NTR^ receptor, while mBDNF exhibits substrate specificity for tyrosine receptor kinase B (TrkB).^3,4^ Interestingly, pro-BDNF signaling through the p75^NTR^ receptor is described to have an apoptotic or pruning effect in neurons,^3^ indicating a yin-yang-like function of the 2 isoforms with recent findings suggesting that the BDNF/pro-BDNF ratio is shifted in neurological conditions, for example, in Alzheimer's.^5^ Brain-derived neurotrophic factor is highly expressed in the central nervous system,^2^ but a significant amount is also present in the circulation,^6,7^ where mBDNF is largely bound to platelets^8–12^ with serum levels 10-100-fold higher than those in plasma.^9,10,13,14^

Brain-derived neurotrophic factor is, however, not exclusively expressed in the brain. Koliatsos et al.^15^ initially identified BDNF mRNA in extracts from rat skeletal muscle tissue, and the myofiber presence of BDNF mRNA in rat muscle was later confirmed using in situ hybridization and electron microscopy.^16^ While BDNF mRNA is readily detectable, the levels and localization of BDNF protein in skeletal muscle tissue are controversial.^17^ Mousavi et al.^18^ reported BDNF protein levels in rat skeletal muscle homogenates to be quite low, ∼0.1 pg mg^−1^ muscle. Further, localization studies on rodent skeletal muscle have shown the presence of BDNF within the myofiber, in satellite cells, in endothelial cells, and in the terminal ends of neurons.^19–21^ Moreover, BDNF mRNA and protein are more highly expressed in oxidative compared to glycolytic rodent muscle.^18,19,22^ However, data on BDNF protein in human skeletal muscle are limited. Specifically, quantifiable data are lacking, and to our knowledge, no attempts have been made to distinguish between pro-BDNF and mBDNF, or to assess whether there is a fiber type-dependent expression.

Exercise increases mRNA and protein levels of BDNF in rat brain.^23,24^ Moreover, studies consistently demonstrate a release of mBDNF from human brain during exercise^8,25,26^ and that exercise increases circulating levels of the neurotrophin.^27^ Matthews et al.^14^ detected mBDNF in human skeletal muscle homogenates and the levels were increased 24 h after endurance exercise. More recently, McKay et al.^28^ reported the presence of BDNF (unspecified isoform) in human satellite cells and that these levels increased, along with whole muscle BDNF mRNA, 24-72 h after exercise. Several exercise-induced myokines and hepatokines have been highlighted as molecular stimulators of exercise-induced brain BDNF production and release.^29,30^ Among these, lactate and β-hydroxybutyrate (β-HB) have gained mechanistic support in rodent brain and in vitro models^31–33^ but their role in humans remain to be elucidated.

The purpose of the present investigation was to quantify the expression of pro- and mBDNF in human skeletal muscle and plasma, and to examine whether physiological interventions (exercise, lactate infusion, 3-day fast) alter their levels.

## Methods

### Human Subjects

Muscle biopsy samples used for the present study were obtained from 3 different ongoing projects with additional objectives. Samples from project 1^34^ were used for antibody validation, for fiber type-specific analysis and for determining the effect of acute exercise and lactate on pro-BDNF and mBDNF levels. Samples from project 1 were obtained from 16 physically active and healthy males (*n* = 8) and females (*n* = 8) with an age, height, and weight of 28 ± 7 years, 173 ± 10 cm, and 69 ± 11 kg, respectively. Samples from project 2 were classified as type I or type II muscle fiber dominant (according to myosin heavy chain sodium dodecyl-sulfate polyacrylamide gel electrophoresis (SDS-PAGE)) and used to evaluate the fiber type-specific expression of pro-BDNF as well as the influence of fasting on plasma levels of β-HB, pro-BDNF, and mBDNF. Samples from project 2^35^ were obtained from 19 physically active and healthy males (*n* = 15) and females (*n* = 4) with an age, height, and weight of 28 ± 5 years, 178 ± 8 cm, and 77 ± 10 kg, respectively. Samples from project 3 were used for the fiber type-specific immunohistochemistry (IHC) analyses and were obtained from 4 physically active and healthy males (*n* = 2) and females (*n* = 2) with an age, height, and weight of 31 ± 5 years, 168 ± 7 cm, and 71 ± 9 kg, respectively. All 3 projects were approved by The Swedish Ethical Review Authority (Project 1 Dnr: 2017/1139-31/4, Project 2 Dnr 2019-02671, and Project 3 Dnr 2019-00381).

### Skeletal Muscle Samples

Muscle samples were collected in the morning after an overnight fast in the resting state. Samples used to study the acute influence of muscle contractile activity were also taken 90 min, 180 min, and 24 h after resistance exercise, with subjects being fasted until 180 min after exercise, as described in ref.^34^ All samples were taken from the vastus lateralis muscle under local anesthesia, using a Bergström needle with applied suction.^36^ After collection, samples were rapidly blotted free from blood and immediately frozen in liquid nitrogen. Samples were stored in −80°C, lyophilized, and carefully dissected free from blood and connective tissue into small fiber bundles, which were subsequently split into aliquots. For the fiber type-specific analyses, type I and type II fibers were isolated from the resting muscle samples from project 3. Following fiber type determination with the THRIFTY method,^37^ all type I and type II fibers from 1 sample were pooled separately (∼100 fibers per sample), weighed, and then prepared for immunoblotting and enzyme-linked immunosorbent assay (ELISA) as described below. In the case of the 4 muscle samples used for IHC, samples were frozen in isopentane chilled in liquid nitrogen for subsequent cryosectioning.

### Sample Preparation

All muscle samples used for immunoblotting and ELISA were homogenized as described in detail in ref.^34^ Briefly, samples were homogenized in a HEPES-based buffer (pH 7.4) containing several protease and phosphatase inhibitors and 1% Triton X-100 as detergent, using a BulletBlender (Next Advance, New York, USA). The homogenates were then rotated and centrifuged to obtain supernatants, of which the protein concentrations were determined. Samples were diluted to a final concentration of 1.5 µg protein µL^−1^ in 4× Laemmli sample buffer (LSB; Bio-Rad Laboratories, Richmond, CA, USA) for immunoblotting, and 0.5 µg µL^−1^ in sterile PBS containing 10% fetal bovine serum (FBS) and 0.02% Tween for ELISA.

For antibody validation purposes, platelet-poor plasma (PPP) and platelet concentrates were prepared. For PPP, whole blood was sampled in EDTA tubes and centrifuged at 3000 × *g* for 10 min, whereafter the top 50% of the plasma obtained was transferred to a new tube. After a second spin at 3000 × *g* for 10 min, the top 75% of the plasma was collected as PPP of which the protein concentrations were determined. For platelet concentrates, whole blood was collected in three 6 mL EDTA-tubes, which were centrifuged at 200 × *g* for 15 min. The plasma obtained (∼10 mL) was collected and pooled in a sterile tube, which was subjected to a second spin at 1000 × *g* for 10 min, which resulted in a platelet-concentrated pellet. The majority of the supernatant was discarded after which 5 mL of sterile PBS was added for washing, followed by an additional spin 1000 × *g* for 10 min. The supernatant was completely removed, and the pelleted platelets were lysed in 2 mL of HEPES-based buffer (as above), after which the protein concentration of the extract was determined. Both the plasma and the platelet extracts were diluted to a final concentration of 1.5 µg µL^−1^ in 4× LSB and HEPES-based buffer. Recombinant proteins and commercially available cell extracts were prepared according to the manufacturer’s instructions.

### Immunoblotting

The immunoblotting process was performed as described in ref.^34^ with minor modifications. The amount of protein loaded in each well is specified below for the antibody validation experiments, while 20 and 2.5 µg of protein were loaded for the whole muscle homogenate and fiber type pool specific analyzes, respectively. Both 18- and 26-well Criterion TGX gradient (4%-20% acrylamide, Bio-Rad Laboratories, Richmond, CA, USA) were used for electrophoresis. Finally, all antibodies were probed on membranes on first use, and no stripping or re-probing were performed. Details about the antibodies used for immunoblotting are presented below.

### Antibody Validation with Immunoblotting

A total of 4 different antibodies were procured for validation purposes. Rabbit polyclonal antibody (NB100-98682) produced with an unspecified immunogen peptide reported to be located in the mature BDNF domain (aa 129-247) was purchased from Novus Biologicals (Abingdon, UK). Rabbit polyclonal antibody (BDNF 19HCLC), reported to be produced with a recombinant protein immunogen for aa 129-247 of human BDNF was purchased from Thermo Scientific (Waltham, MA, USA). Mouse monoclonal antibody (pro-BDNF 9C1), reported to be raised against recombinant human pro-BDNF was from Santa Cruz Biotechnology (Dallas, TX, USA). Finally, a mouse monoclonal antibody supplied in the pro-BDNF ELISA kit (DY3175) from R&D Systems (Abingdon, UK) was reported to have 1.4% cross-reactivity with mBDNF was used.

In the first round of validation, the following samples were loaded: (1) 22 µg human skeletal muscle protein; (2) 20 µg human platelet protein extract (known to be highly enriched with mBDNF); (3) 20 µg human plasma protein (known to be enriched with pro-BDNF); (4) human type I fiber pool (4 µg protein): (5) human type II fiber pool (4 µg protein); (6) 0.25 ng recombinant mBDNF (from #DBD00, R&D Systems); and (7) 0.25 ng recombinant pro-BDNF (from #DY3175, R&D Systems). Antibodies were diluted 1:1000 (Novus and Thermo), 1:500 (Santa Cruz Biotechnology), or 0.5 µg mL^−1^ (R&D Systems) in TBS supplemented with 2.5% nonfat dry milk and 0.1% Tween-20 (TBS-T milk).

In the second round of validation, using only the Novus antibody, we used the following samples: (1) 25 µg plasma protein mixed from 8 subjects from project 1, (2) 20 µg human platelet protein extract, (3) 20 µg human skeletal muscle protein, (4) 20 µg of mouse soleus protein, (5) 20 µg of mouse extensor digitorum longus (EDL) protein, (6) 35 µg protein from HeLa cell lysate (negative control), (7) 0.1 µg Mouse IgG (negative control), and (8) 1 ng recombinant pro-BDNF.

Finally, we validated the Abcam #ab108319 Ab for the detection of mBDNF in human skeletal muscle using the following samples: (1) human skeletal muscle homogenate 40 µg protein; (2) human skeletal muscle homogenate 20 µg protein; (3) human type I fiber pool 25 µg protein; (4) human type II fiber pool 25 µg protein; (5) human plasma protein 15 µg of protein; (6) human plasma protein 30 µg of protein; (7) human platelet extract 4 µg protein; (8) human platelet extract 8 µg protein; (9) human platelet extract 12 µg protein; (10) HeLa cell lysate (Santa Cruz Biotechnology sc-2200) 35 µg protein, serving as a negative control lysate; (11) mouse IgG 0.1 µg, serving as a negative control; (12) recombinant BDNF 0.25 ng (from #DBD00, R&D Systems).

### Enzyme-Linked Immune Absorbent Assay

Muscle levels of pro-BDNF (pg mg^−1^ dry muscle) were quantified using the Human Pro-BDNF DuoSet ELISA kit (DY3175) combined with the DuoSet Ancillary Reagent Kit 2 (DY008) from R&D Systems. Assays were run according to the manufacturer’s instructions with 2 modifications. Samples and standards were diluted in sterile PBS with 10% FBS + 0.02% Tween-20, and then incubated in the coated plate overnight (16 h) at 4°C. All muscle samples were diluted to a final concentration of 0.5 µg µL^−1^ with 50 µg of protein (100 µL) loaded in each well. The assays were performed with samples and standards in duplicate. A representative standard curve had the following concentration (pg mL^−1^) and optical density at 450 nm in brackets: 0 (0.156), 78 (0.307), 156 (0.414), 313 (0.623), 625 (0.937), 1250 (1.452), 2500 (2.143), and 5000 (2.847). The optical density for 50 µg of muscle protein generally ranged from 0.400 to 0.600 for the corresponding standard curve.

### Immunohistochemistry

From 4 muscle biopsy samples, 7 µm thick cross-sections were cut using a cryostat (Leica CM1950) and mounted together on a microscope glass slide (VWR International). Slides were then air-dried at room temperature (RT) to remove excess water and then stored in −80°C until staining procedures commenced. For pro-BDNF staining, slides were fixed in 4% paraformaldehyde for 10 min, washed in phosphate buffered saline (PBS) (3 × 5 min) and blocked for 2 h at RT in a solution containing 5% normal goat serum (NGS), 2% bovine serum albumin (BSA), 5% FBS, and 0.02% Triton X-100. Next, sections were incubated overnight (4°C) with primary antibodies against pro-BDNF (1:200, 9C1), MyHC-I (1:200; BA-F8), and dystrophin (1:200; MANDYS) diluted in 1% BSA. The antibody against pro-BDNF was purchased from Santa Cruz Biotechnology (9C1), and the antibodies against MyHC-I and dystrophin were both purchased from the Developmental Studies of Hybridoma Bank (DSHB), USA. The next day, after washes in PBS (3 × 5 min), sections were incubated for 1 h with species and subclass-specific secondary antibodies (1:200, Alexa Fluor, Invitrogen, USA) diluted in 1% NGS, before being mounted with Prolong Gold Antifade Reagent (Invitrogen, USA).

Stained sections were captured using a widefield fluorescent microscope (Celena^®^ S, Logos Biosystems, South Korea), and digital pictures were processed using the built-in image analysis software. Images were captured at 10× magnification and image parameters were kept constant across all subjects, including light (100), gain (18), and exposure time (40 ms). Image analysis was carried out using ImageJ (National Institutes of Health, USA) and from each biopsy an average of 39 ± 6 and 41 ± 5 type I and type II fibers were included, respectively. Mean pixel intensity of the pro-BDNF staining in different fiber types was obtained by manually encircling the border of individual fibers in the greyscale image. These fibers were then categorized as type I and type II fibers using the overlay image of dystrophin and MyHC-I. The final mean pixel intensity of each fiber was obtained after the inherent background staining intensity had been subtracted and this was measured in a section stained without the application of primary antibodies.

### Brain-Derived Neurotrophic Factor mRNA Expression

Whole muscle mRNA expression analysis was performed as described in ref.^38^ The primers used for quantitative polymerase chain reaction (qPCR) were as follows, forward: *AGCCCTGTATCAACCCAGAA* and reverse: *CAATGCCAACTCCACATAGC*.

The fiber type-specific RNA-seq dataset of Rubenstein et al.^39^ was downloaded from the gene expression omnibus (GEO) repository under accession number GSE130977. This dataset consists of RNA-seq data of pools of type I or type II fibers from m. vastus lateralis biopsies of 9 healthy, older men. For full details on generation of this dataset, we refer to the original publication.^39^ Raw counts were normalized with DESeq2 to allow for between-sample comparisons.^40^ First, normalized counts for MYH2 (ENSG00000125414, type II fibers) and MYH7 (ENSG00000092054, type I fibers) were extracted and assessed for each fiber pool as purity quality control. Based on this analysis, the fiber pools of 1 participant were excluded for further analysis. Next, normalized counts of BDNF (ENSG00000176697) were extracted and compared between type I and type II fiber pools within each participant.

### Statistics

Data were analyzed using TIBCO Statistica 13 for Windows (TIBCO Software Inc., Palo Alto, CA, USA). Data are presented as mean ± SEM or individual values, unless otherwise noted. The normal distribution of variables was explored prior to execution of tests with histograms and Shapiro-Wilks test of normality. For the immunoblotting, data on the effect of exercise on pro-BDNF expression were skewed, and after log-transformations all data were deemed acceptable for parametric statistical tests. Student’s unpaired *t*-test was employed to determine differences in pro-BDNF expression between type I and type II dominant groups, while a paired *t*-test was used between type I and type II fiber pools and for the fiber type-specific RNA-seq data. A multivariate analysis of variance was used to asses differences between type I and type II fibers with IHC. Pearson’s correlation was performed to analyze the relationship between type I fiber content and pro-BDNF expression. A 2-way repeated measures analysis of variance (ANOVA) with time and infusion as factors was used for data analysis for plasma lactate, β-HB, pro-BDNF, mBDNF as well as for muscle mRNA and protein expression of BDNF. A 2-way repeated measures analysis of variance (ANOVA) with time and fiber-type dominance as factors was used for data analysis for plasma lactate, β-HB, pro-BDNF, and mBDNF. Tukey’s Honest Significance Difference tests were performed if a significant main effect or interaction appeared. Statistical significance was set at *P* < 0.05. The statistical data that support the findings of this study are available in the Statistics Supplementary file.

## Results

### Detection of Pro-BDNF and mBDNF in Human Skeletal Muscle

**Figure 1.**
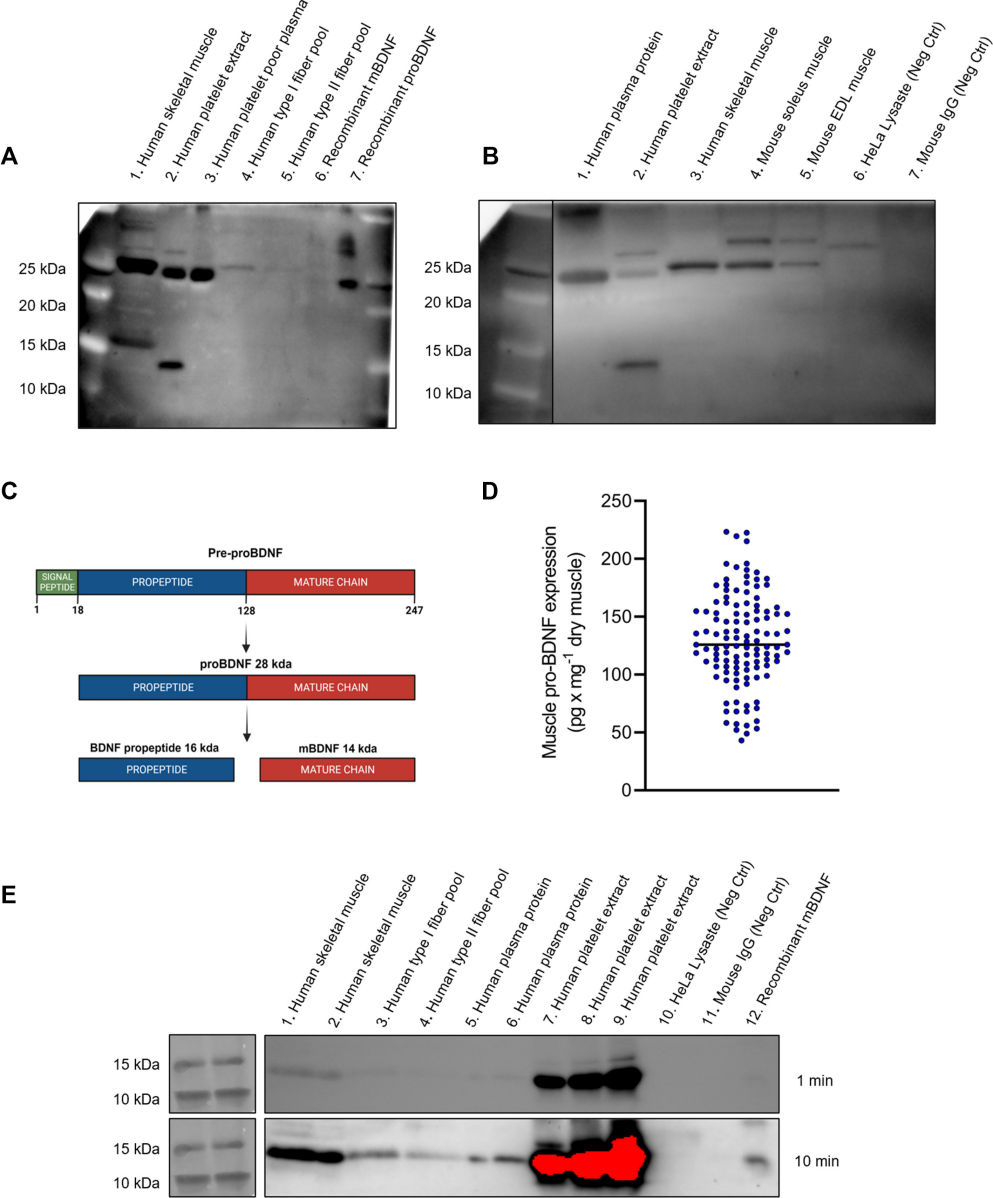
Antibody validation and detection of pro-BDNF in human skeletal muscle. Validation of the Novus (NB100-98682) antibody in its ability to detect pro-BDNF in different samples. (A) The gel is loaded according to the following: (1) human skeletal muscle homogenate 22 µg protein; (2) human platelet extract 20 µg protein; (3) human platelet poor plasma 20 µg protein; (4) human type I fiber pool 4 µg protein; (5) human type II fiber pool 4 µg protein; (6) recombinant mBDNF 0.25 ng; and (7) recombinant pro-BDNF 0.25 ng. The antibody is able to detect pro-BDNF at 28 kDa. The antibody also detects mBDNF at 14 kDa, found in the platelet extract that is known to be highly enriched with mBDNF. (B) In this gel, blotted with NB100-98682: is (1) human plasma samples with 25 µg of protein loaded; (2) human platelet extract 20 µg protein; (3) human skeletal muscle homogenate 20 µg protein; (4) mouse Soleus muscle homogenate 20 µgprotein; (5) mouse EDL muscle homogenate 20 µg protein; (6) HeLa cell lysate (Santa Cruz Biotechnology sc-2200) 35 µg protein, serving as a negative control lysate; and (7) mouse IgG 0.1 µg, serving as a negative control. (C) Schematic overview of BDNF metabolism. (D) Skeletal muscle levels of pro-BDNF (pg mg^−1^ dry muscle) for all the 117 muscle samples analyzed in the study. (E) Validation of the Abcam (ab108319) antibody to detect mBDNF in different samples: (1) human skeletal muscle homogenate 40 µg protein; (2) human skeletal muscle homogenate 20 µg protein; (3) human type I fiber pool 25 µg protein; (4) human type II fiber pool 25 µg protein; (5) human plasma protein 15 µg of protein; (6) human plasma protein 30 µg of protein; (7) human platelet extract 4 µg protein; (8) human platelet extract 8 µg protein; (9) human platelet extract 12 µg protein; (10) HeLa cell lysate (Santa Cruz Biotechnology sc-2200) 35 µg protein, serving as a negative control lysate; (11) mouse IgG 0.1 µg, serving as a negative control; and (12) recombinant mBDNF 0.25 ng.

We first determined the expression of pro-BDNF in human skeletal muscle using immunoblotting. To that end, we compared 4 commercially available antibodies and validated their ability to detect pro-BDNF (details about these antibodies can be found in the “Methods” section). In the first round of validation, we used human skeletal muscle protein, human platelet protein extract (known to be highly enriched with mBDNF), human plasma protein (known to be enriched with pro-BDNF), human type I and type II skeletal muscle fiber pools, recombinant mBDNF, and pro-BDNF. The results are presented in Supplementary Figure S1. All antibodies detected a band at 28 kDa (pro-BDNF) in the muscle, platelet, and plasma samples. An antibody from Novus (NB100-98682) also detected pro-BDNF in the type I and type II fiber pools, which were loaded in low-protein contents. All antibodies also detected a band at ∼16 kDa in the human muscle sample, which according to the size and controls should not be mBDNF but could be the BDNF propeptide. Recombinant pro-BDNF was identified at the expected molecular weight by the antibody from Novus, which was the only antibody that identified mBDNF at 14 kDa in the platelet protein extract and also gave a weak signal for recombinant mBDNF. Collectively, the antibody from Novus (Figure 1A) provided valid results with a high signal to noise ratio and was therefore used for the subsequent immunoblotting experiments.

In the second round of validation, using only the Novus antibody, we added mouse muscle homogenates as well as 2 negative controls. The results from the second round of validation are presented in Figure 1B. The result confirmed previous observations and showed that pro-BDNF could also be detected in mouse muscle using this antibody. Importantly, both negative controls gave no signal for pro-BDNF. See Figure 1C for an overview of BDNF processing.

We then quantified the levels of pro-BDNF in human skeletal muscle. Using ELISA, we found that the content of pro-BDNF ranged from 42-248 pg mg^−1^ dry muscle (132 ± 44) in 39 healthy young subjects (Figure 1D).

In parallel, we also attempted to quantify the levels of mBDNF in human skeletal muscle samples using ELISA. Despite loading up to 350 µg of protein (representing approximately 1 mg of dry muscle) the signals acquired were between the blank and the lowest standard at 31 pg mL^−1^. This would suggest a maximal content of 1-2 pg mg^−1^ dry muscle. The much lower levels of mBDNF compared to pro-BDNF in human skeletal muscle are in agreement with the inability to detect mBDNF with immunoblotting in our initial validations. Following a literature search, we identified a candidate antibody for detection of mBDNF (Abcam ab108319). Immunoblotting with that antibody resulted in detection of mBDNF in human skeletal muscle, a clear detection in human platelet extracts, as well as detection of recombinant mBDNF following prolonged exposure (Figure 1E).

### Higher Expression of Pro-BDNF in Type I Fibers

That pro-BDNF expression may be fiber type-dependent was initially tested during antibody validation, where the type I fiber pool from 1 subject had a higher expression than the type II fiber pool (Figure 1A). This was further supported by the higher expression in mouse soleus compared to EDL muscle (Figure 1B).

**Figure 2.**
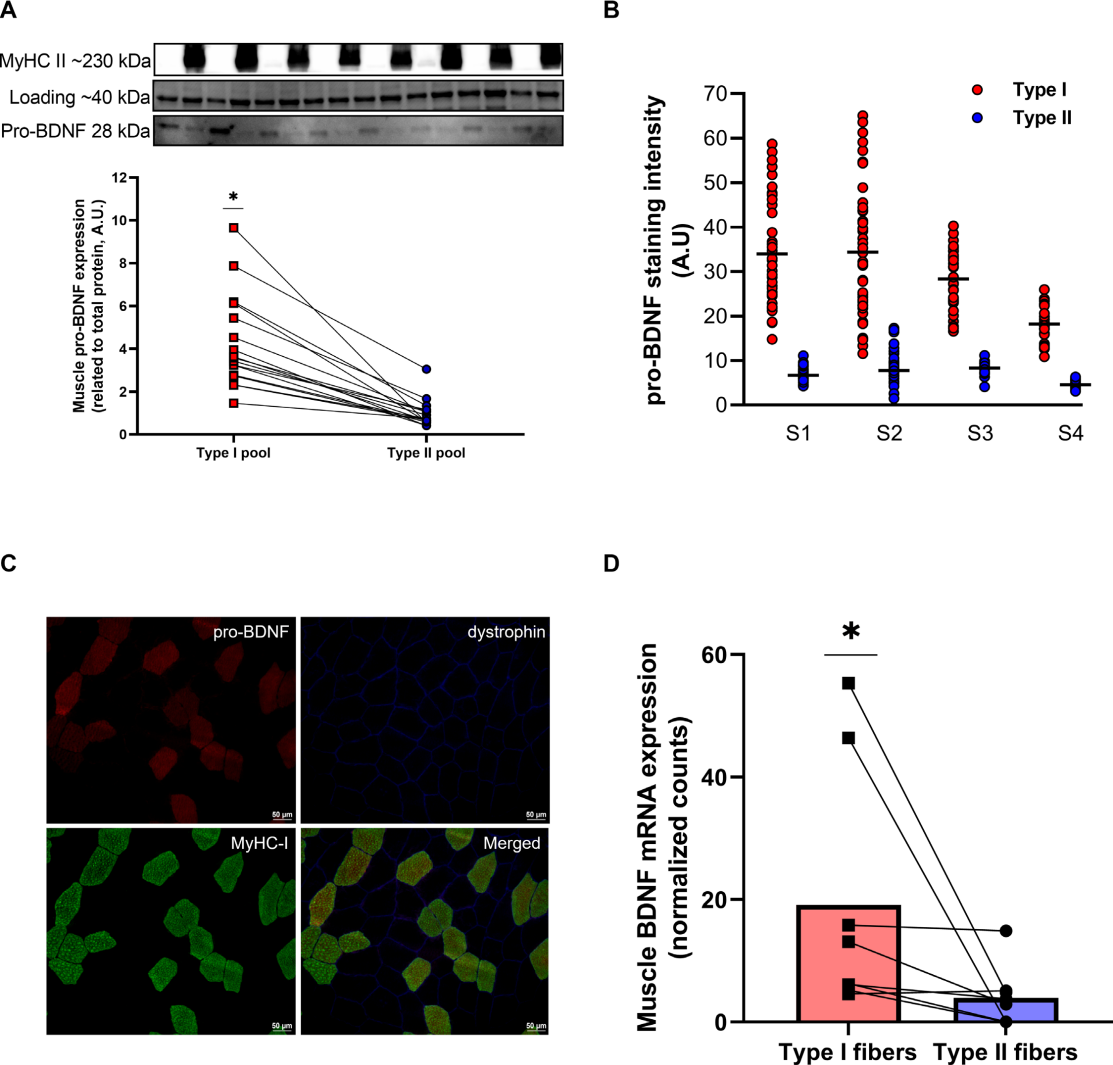
Fiber type-specific expression of pro-BDNF in human skeletal muscle. (A) Muscle expression of pro-BDNF in pools of type I and type II fibers determined with immunoblotting (*n* = 16). Representative blots for pro-BDNF at 28 kDa, the total protein stain at ∼40 kDa (actin) and MyHC-II (type II myosin) for 8 sets of pools are provided above the graph. The *P < 0.05 vs. type II pools. (B) Quantification of pro-BDNF signal intensity (arbitrary units) in type I (red dots) and type II (blue dots) fibers for *n* = 4. Each dot represents the signal intensity in 1 fiber. *P < 0.05 vs. type II fibers. (C) Representative images from the IHC stainings, pro-BDNF (red, 595 nm), dystrophin (blue, 388 nm), MyHC-I (green, 488 nm) and merged image of pro-BDNF (red), MyHC-I (green), and dystrophin (blue). All images were captured at 10× magnification. Scalebar is 50 µm. (D) Fiber type-specific mRNA expression of BDNF. Fiber type-specific RNA-seq dataset from the GEO repository under accession number GSE130977 was used. The dataset consists of RNA-seq data from pure pools of type I or type II fibers from m. vastus lateralis biopsies of 8 healthy, older men. Normalized counts of BDNF (ENSG00000176697) were extracted and compared between type I and type II fiber pools within each participant. *P < 0.05 vs. type II pools.

Next, we determined pro-BDNF expression in pools of type I and type II fibers from 16 subjects. Here, we found a 4.4-fold higher expression in the type I pools (*P* < 0.001, Figure 2A). Furthermore, fiber-type dependence was also investigated with IHC. Using muscle samples from 4 young healthy individuals we found the pro-BDNF expression to be 4.2-fold higher in type I compared to type II fibers (*P <* 0.001, Figure 2B and C).

We then assessed whether muscle fiber-type dependency for BDNF is also present at the transcriptional level, since the BDNF gene codes for pro-BDNF, which is subsequently cleaved to mBDNF. For this purpose, we prepared fiber type-specific pools from 8 subjects from project 1, where each pool contained 10 type I or type II fibers (approximately 25 µg). Following RNA extraction, we attempted a qPCR approach to determine levels of BDNF. However, these experiments were not successful owing to the limited sensitivity of the assay. We then turned to the existing fiber type-specific RNA-seq dataset from Rubenstein et al. (2020). Ad hoc analysis of their dataset revealed a 5-fold higher BDNF expression in type I compared to type II fibers (*P* < 0.05, 2D), which can be contrasted with the 4.2 to 4.4-fold difference at the protein level.

**Figure 3.**
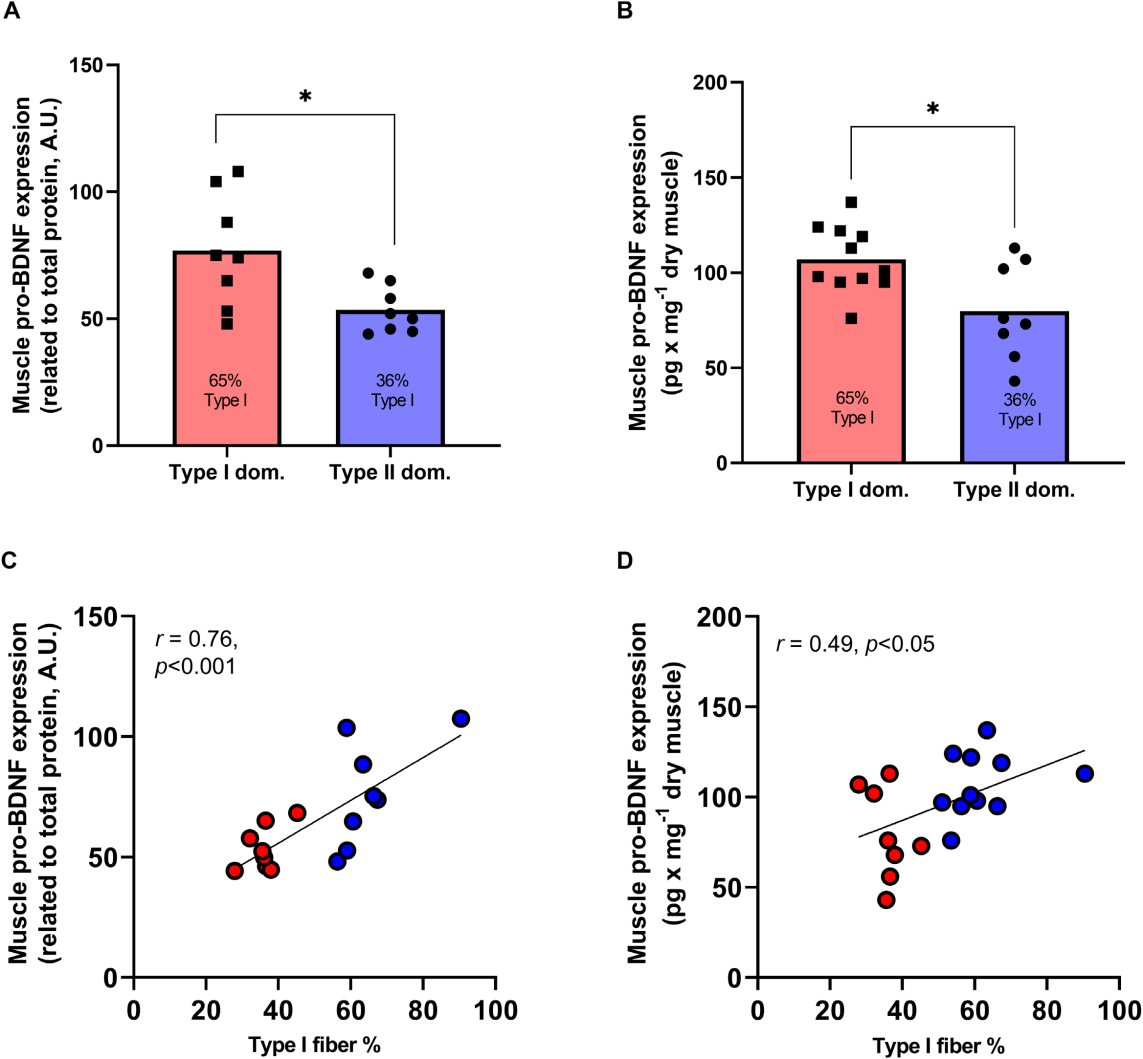
Skeletal muscle expression of pro-BDNF in 2 groups of subjects with type II or type I fiber dominant (*n* = 16) content determined with immunoblotting (A) or ELISA (B). The type II dominant group has a mean of 36% type I fibers, while the type I dominant group has a mean of 65% type I fibers in the biopsies taken from the vastus lateralis. The asterisk represents *P* < 0.05 compared to type II dominant. Correlation between skeletal muscle pro-BDNF expression and type I fiber content (*n* = 16) determined with immunoblotting (C) or ELISA (D).

We then compared pro-BDNF expression in biopsies from subjects with a high content of type I fibers (65% ± 11%, determined by SDS-Page) and low content of type I fibers (36% ± 5%). Immunoblotting yielded a 43% higher expression in the type I dominant group (Figure 3A, *P* < 0.01), while ELISA showed a 35% higher expression in the type I dominant group (*P* < 0.05, 3B). We also found significant positive correlations between pro-BDNF expression and type I fiber content using both immunoblotting (*r* = 0.76, *P* < 0.001, Figure 3C) and ELISA (*r* = 0.49, *P* < 0.05, 3D).

Taken together, these analyses demonstrate that: (1) pro-BDNF is the sole significant isoform in human skeletal muscle; (2) pro-BDNF is expressed primarily in type I muscle fibers; (3) the differential expression of the protein is associated with comparable differences in mRNA levels between fiber types; and (4) the fiber type-specific pro-BDNF expression also appears in whole muscle levels in individuals with varying fiber type proportions.

### Venous Lactate Infusion Augments Exercise Induced Plasma mBDNF Levels

**Figure 4.**
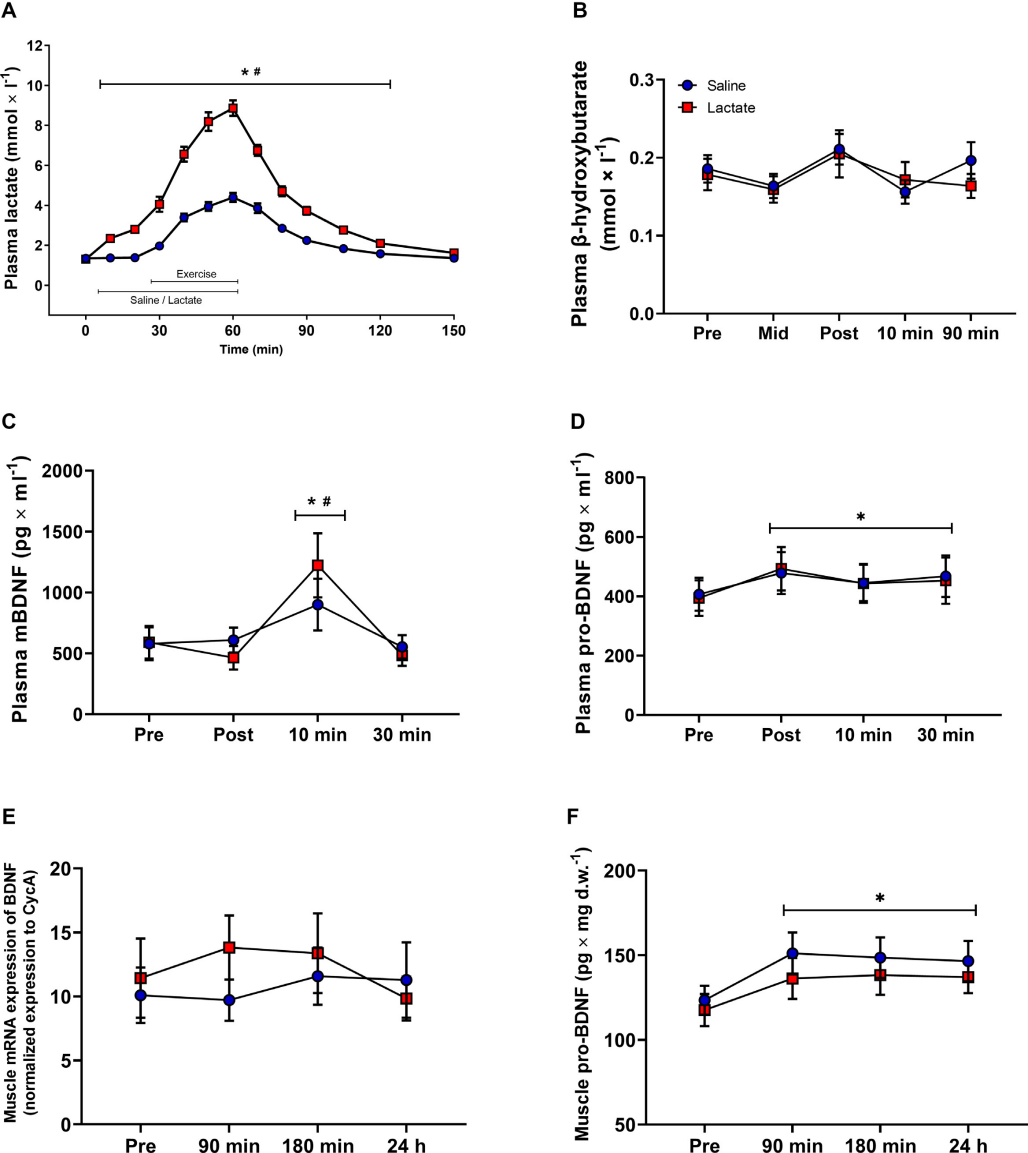
Influence of exercise and lactate infusion of plasma and muscle BDNF. Plasma levels of (A) lactate, (B) β-hydroxybutarate, (C) mBDNF, and (D) pro-BDNF in samples from project 1. Plasma samples were taken before, during and after exercise performed with infusion of sodium lactate (Lactate) or saline (control). (E) Muscle mRNA expression of BDNF and (F) protein expression of BDNF (pg mg^−1^ dry muscle) in muscle samples taken at rest (Pre) and 90 min, 180 min and 24 h after exercise in project 1. Blue symbols represent exercise with Saline infusion and red symbols represent Lactate infusion. Values presented are means ± SEM for *n* = 16. The *P < 0.05 vs. rest (Pre); # *P* < 0.05 vs. Saline.

Nonhuman research indicates that lactate is a molecular trigger of brain BDNF production.^33^ Therefore, we studied the effects of acute resistance exercise, with or without venous infusion of sodium lactate,^34^ on BDNF metabolism. Exercise with saline infusion (placebo) resulted in peak plasma lactate levels of 4.4 ± 0.9 mmol L^−1^, whereas peak values of 8.9 ± 1.6 mmol L^−1^ were achieved during lactate infusion (Figure 4A, *P* < 0.001). Plasma levels of β-HB were not altered during the trials (Figure 4B).

In the Saline-trial, exercise induced a 55% increase in plasma levels of mBDNF (Figure 4C, *P* < 0.05 vs. Pre) 10 min after completion of exercise. Exercise with lactate infusion resulted in an 115% increase in plasma mBDNF levels compared to the value at rest, resulting in 35% greater levels compared to the Saline-trial at that time point (Figure 4C, *P* < 0.05 Time and Trial Interaction). Exercise resulted in a 18%-25% increase in plasma levels of pro-BDNF (Figure 4D, *P* < 0.01 vs. Pre), which was not affected by lactate infusion.

### Muscle Contractions Stimulate Pro-BDNF Expression in Human Skeletal Muscle

We also assessed the muscle expression of BDNF mRNA, mBDNF, and pro-BDNF. Using qPCR, we found that skeletal muscle expression of BDNF was not sensitive to muscle contractions with or without lactate infusion (Figure 4E). Using immunoblotting there was no change in mBDNF or pro-BDNF expression at any of the 3 time points studied after exercise (data in Supplement). However, using ELISA we found a ∼20% increased protein expression of pro-BDNF at 90 min after exercise, which then remained at the same level until 24 h after exercise (Figure 4F, *P <* 0.05 vs. Pre). Lactate infusion had no effect on muscle pro-BDNF expression. Muscle levels of mBDNF were never detectable using ELISA (data not shown).

### Fasting Increases Circulating Levels of Pro-BDNF

**Figure 5.**
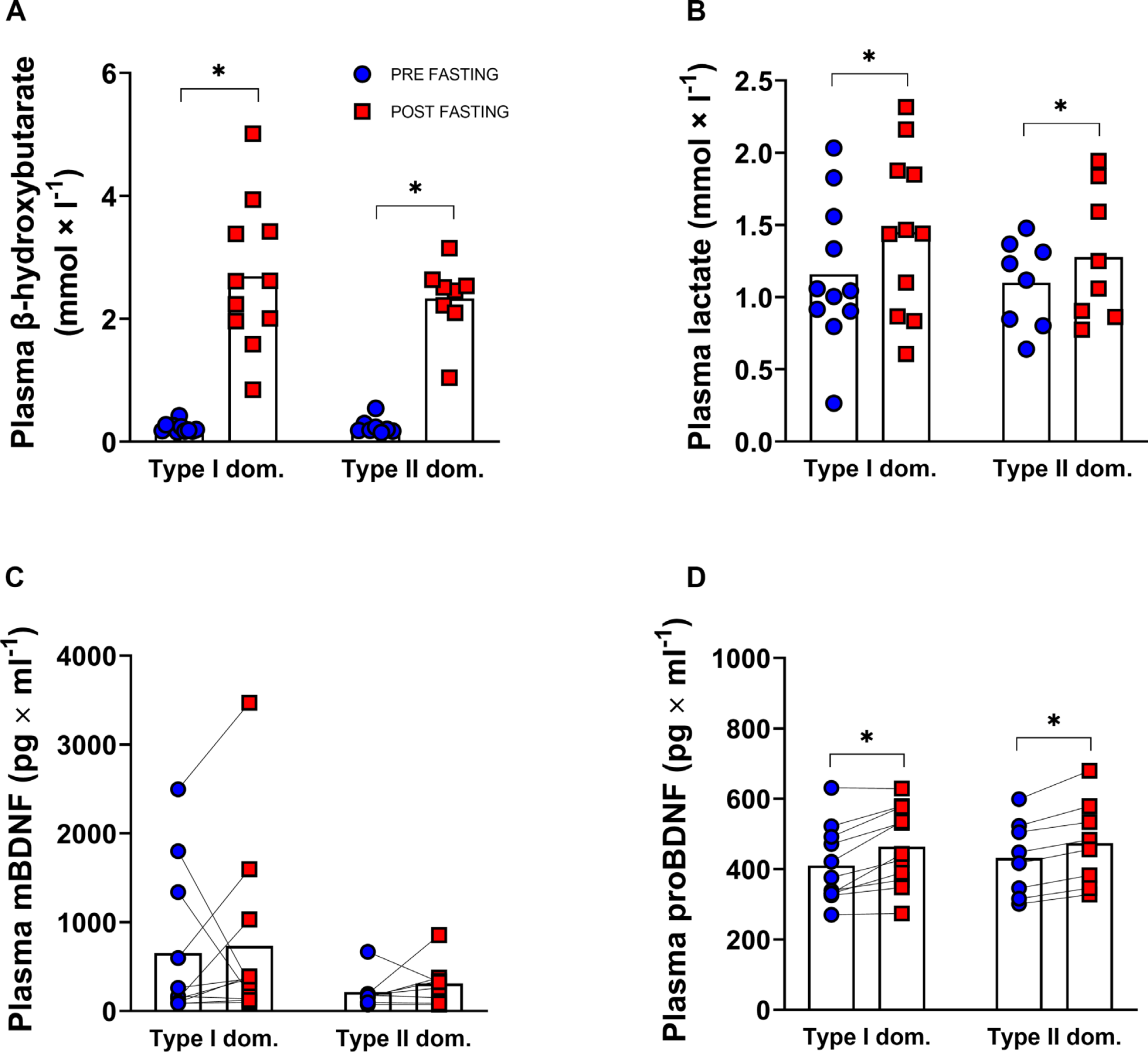
Effects of a 3-day fast on plasma metabolites and BDNF-isoforms. Plasma levels of (A) β-hydroxybutarate, (B) lactate, (C) mBDNF, and (D) pro-BDNF from in samples from project 2. Plasma samples were taken before and after a 72 h fasting period in subjects with a type I muscle fiber dominance (*n* = 11) or a type II muscle fiber dominance (*n* = 8). Each symbol is an individual data point, where dot symbols represent samples taken before fasting and square symbols samples taken after fasting. The *P < 0.05 pre vs. post fasting.

It is well established that the ketone body β-HB is a potent activator of mBDNF production in brain.^32^ A well-established physiological intervention to induce marked increases in circulating β-HB levels in humans is fasting.^35^ Therefore, we investigated the effects of a 3-day fast on circulating levels of pro-BDNF and mBDNF, as well as the influence of the subjects’ muscle fiber type. The 3-day fast elevated plasma β-HB ∼10-fold to an average of 2.5-2.7 mmol l^−1^ (Figure 5A, *P <* 0.001), in both type I and type II dominant subjects, and had a minor effect on plasma lactate levels (Figure 5B, *P <* 0.05). Plasma levels of mBDNF did not differ significantly between type I and type II dominant individuals and did not change in response to the 3-day fast (Figure 5C). In contrast, a 10%-13% increase in plasma pro-BDNF levels were noted after the 3-day fast (Figure 5D, *P <* 0.05), but the response was similar regardless of fiber type distribution.

## Discussion

A detailed analysis of pro-BDNF and mBDNF was performed in human muscle and plasma at rest and after several physiological interventions. The major findings are that: (1) pro-BDNF is expressed in human skeletal muscle, whereas mBDNF is not detectable; (2) pro-BDNF protein and mRNA are expressed primarily in type I muscle fibers; (3) resistance exercise results in increases in pro-BDNF and mBDNF in plasma; (4) infusion of sodium lactate during exercise increases plasma levels of mBDNF versus placebo, but does not affect plasma levels of pro-BDNF; and (5) 3-day fasting does not alter plasma levels of mBDNF but results in a small increase in plasma pro-BDNF.

A key purpose of the present study was to investigate the expression of pro-BDNF and mBDNF protein in human skeletal muscle. Following a series of antibody validations, we were clearly able to detect pro-BDNF, but observed only low or nondetectable levels of mBDNF in human skeletal muscle homogenates using immunoblotting. We also quantified the amount of pro-BDNF in human skeletal muscle to be 42-248 pg mg^−1^ dry muscle with ELISA analyses. The inability to detect mBDNF could not be ascribed solely to insufficient sensitivity of the antibodies, since ELISA analyses, which clearly detected mBDNF in plasma, only yielded a maximal content of 1-2 pg mg^−1^ dry weight of mBDNF in muscle. Assuming an extracellular water content of 0.5 µL mg^−1^ dry weight^41^ and that the plasma concentration (500 pg mL^−1^; see Figure 4) is equal to that in extracellular water, it can be calculated that ∼0.2 pg mg^−1^ dry weight mBDNF is of extracellular origin. However, considering that the serum concentration of mBDNF is ∼10-fold higher than that in plasma^9^ (owing to the presence of platelets), the muscle content of mBDNF that is of extracellular origin would amount to ∼2 pmol mg^−1^ dry weight. Taken together, the data indicate that there is no detectable mBDNF in human skeletal muscle cells, using ELISA and immunoblotting, under physiological conditions. Similar calculations for the extracellular contribution of pro-BDNF to the muscle content also indicate ∼0.2 pg mg^−1^ dry weight. The latter calculation indicates that virtually all of the pro-BDNF in the muscle analyses originates within the muscle.

We also found a higher expression of pro-BDNF in type I dominant muscle using both immunoblotting and ELISA. Moreover, determination of pro-BDNF in pooled type I and II muscle fibers, using both immunoblotting and IHC, yielded values that were >4-fold higher in type I versus type II fibers. Consistent with these findings was a 5-fold higher expression of pro-BDNF mRNA levels in type I versus type II fibers. Therefore, it appears that a higher rate of transcription contributes to the higher pro-BDNF values in type I muscle fibers. Taken together, the results indicate that a potential release of BDNF from skeletal muscle during exercise is likely in the form of pro-BDNF and not mBDNF. Additionally, type I muscle fibers, which are the primary fibers recruited during submaximal exercise, are likely the major source of an exercise-mediated release of pro-BDNF from muscle. The increase in circulating mBDNF during exercise is likely to, at least partly, derive from the cleavage of pro-BDNF by extracellular proteases.^3^ The observation that the increase in plasma pro-BDNF precedes the increase of mBDNF (Figure 4C and D) is consistent with this explanation.

Earlier studies have implicated lactate in the production and release of mBDNF from the brain. To assess the role of lactate in BDNF metabolism during exercise, we infused sodium lactate prior to and during exercise. This resulted in a significant increase in plasma mBDNF compared to the increase during exercise in the saline trial. Noteworthy, however, the additional increase in plasma mBDNF with lactate infusion was not associated with a further increase in plasma pro-BDNF (Figure 4C and D). One possible explanation for this observation is that during exercise lactate induces a direct release of mBDNF from the brain, either directly or via cleavage of pro-BDNF by intracellular convertases. In this scenario, lactate would be affecting the brain and not skeletal muscle. Future studies are required to address this issue. We also observed that acute exercise resulted in an increase in pro-BDNF protein in muscle at 1.5-24 h post-exercise. This increase was not associated with a change in pro-BDNF mRNA levels, nor was it affected by lactate infusion. The latter findings suggest that exercise-induced increases in translational are involved in the increase in pro-BDNF protein levels during recovery from exercise. The lack of effect of lactate infusion on muscle pro-BDNF protein levels is consistent with the idea that lactate is affecting plasma mBDNF levels by a mechanism that does not involve skeletal muscle (see above).

The mRNA expression of BDNF in the myofiber was convincingly shown by Liem et al.^16^ using in situ hybridization combined with electron microscopy in rat soleus muscle. This finding has been replicated in rat, mouse, and human skeletal muscle using more conventional techniques.^14,18,22,28,42^ In contrast, assessment of protein levels of BDNF has been problematic, particularly concerning the distinction between pro-BDNF and mBDNF. When distinguishing between the isoforms, it is important to consider methodological aspects. Antibodies directed to sequences in the mature domain (aa 129-247) will also detect pro-BDNF, if the mature domain remains bound to the pro-domain. Thus, immunoblotting holds an advantage for detecting different isoforms as detection is based on not only antigen sequence but also molecular weight. However, most studies in the field have not reported molecular weights, provided full-length blots, or validated the antibodies used for immunoblotting. A drawback of immunoblotting is that it generates semiquantitative data. For quantification of BDNF levels, use of ELISA is preferable, but accuracy depends on the specificity of the antigen sequence when the protein is in the native state. Finally, interpretation of previous data is complicated by the fact that some earlier studies used antibodies and ELISA kits that are no longer available.

Using ELISA, Mousavi et al.^18^ quantified BDNF protein levels to be ∼0.1 pg mg^−1^ wet muscle in mouse EDL and Soleus. The BDNF isoform was not specified, but based on the kit used and the protein concentration generated it was likely mBDNF specific. Depending on the homogenization protocol, the concentration would correspond to about 2-8 pg mL^−1^, which lies around the detection limit for commercially available ELISA kits directed toward mBDNF. To our knowledge, only 3 studies have investigated the expression of BDNF (pro-BDNF and/or mBDNF) in human skeletal muscle. First, Matthews et al.^14^ showed the presence of mBDNF in human skeletal muscle using immunoblotting and detected increased protein levels 24 h after exercise. This report did not provide any validation of the mBDNF detection, and used an antibody that is no longer available. Next, McKay et al.,^28^ using an mBDNF target antibody with IHC, detected BDNF in human muscle satellite cells. Finally, Máderová et al.^43^ reported the presence of both mBDNF and pro-BDNF in aging human skeletal muscle using immunoblotting. However, these authors reported protein bands at the wrong location for both isoforms and provided no validation of their approach. Considering the results of the present study, and the discussion above, we suspect that those studies that reported the presence of mBDNF protein in human skeletal muscle were reporting the presence of pro-BDNF, another (nonspecific) protein, or extracellularly derived mBDNF. Using immunoblotting, we acquired a robust signal for pro-BDNF in human skeletal muscle samples at the estimated molecular weight. These data were confirmed with platelet-derived, plasma-derived, and recombinant pro-BDNF as positive controls. With ELISA we quantified pro-BDNF levels in human skeletal muscle. A clearly detectable signal for pro-BDNF was also found with IHC, where the best signal was acquired with the Santa Cruz Biotech (9C1) antibody, which gave a valid but weak signal with immunoblotting. Collectively, our data demonstrate that pro-BDNF is clearly expressed in human skeletal muscle and is the only significant isoform of BDNF in this tissue.

Previous reports indicate that the mRNA and protein expression of BDNF (unspecified isoform) are higher in more oxidative skeletal muscle compared to glycolytic rodent muscle.^18–20^,^22^ In contrast, it was recently shown in mouse muscle that BDNF knockout induces a shift to a slow fiber type, while BDNF overexpression (both isoforms) mediates a fast (glycolytic) fiber type shift.^42^ The latter study, however, did not analyze fiber type-specific BDNF expression in wild-type mice. To our knowledge, there are no previous data on fiber type-specific expression of pro-BDNF in any species, including humans. Here we analyzed muscle samples from type I and type II fiber dominant individuals as well as fiber type-specific pools and utilized a combined immunoblotting and ELISA approach to determine pro-BDNF expression. These analyses were coupled with fiber type-specific expression of pro-BDNF using IHC. Thus, using multiple approaches, our data demonstrated a clear type I fiber dominant expression of pro-BDNF. The different methodological approaches yielded varying degrees of differences in pro-BDNF expression between fiber types but were across all analyses 4-fold higher in type I fibers. Our ad hoc analysis of an existing fiber type-specific RNAseq dataset also supported higher BDNF expression in type I oxidative fibers. In summary, our data are consistent with previous observations of higher BDNF expression in oxidative muscle, which has been suggested to involve control of fatty acid metabolism,^14^ but the function of the fiber type-dependent expression of pro-BDNF in human skeletal muscle requires further investigation.

It is well established that exercise induces an acute and chronic increase in circulating mBDNF in humans.^27,44^ Additionally, high intensity during exercise induces increased levels of blood mBDNF and these correlate with blood lactate levels.^13,45^ Recently, evidence for lactate-induced BDNF expression in mouse brain was provided,^33^ but at present experimental support for this mechanism in humans is lacking. Our data demonstrate that lactate infusion in humans augments exercise-induced plasma levels of mBDNF, but not pro-BDNF. We can at this point not decipher whether this exercise-induced increase in mBDNF is derived from brain, muscle, or platelet, but our data are nevertheless in agreement with El Hayek et al.^33^ Considering the discussion above, it is possible that submaximal exercise primarily results in pro-BDNF release from skeletal muscle, whereas high-intensity exercise will result in higher lactate levels and stimulate a release of mBDNF from the brain.

There is ample evidence that β-HB stimulates BDNF expression in rodent brain.^31,32,46^ Fasting markedly increases synthesis of β-HB. Consistent with this is the observation that fasting studies in animals result in elevated levels of circulating BDNF.^47^ In humans, fasting results in larger increases in plasma β-HB than in rodents.^48^ Our data showed that a 3-day fast, resulting in a 10-fold increase in plasma β-HB, had no impact on plasma mBDNF but induced a small but significant increase in plasma pro-BDNF. The concomitant pro-BDNF and β-HB increase is in line with recent data by Norgren et al.^49^ who showed that fatty acid-induced β-HB levels correlated with circulating pro-BDNF in humans. The reason for the lack of change in mBDNF in the present study is not clear. Nevertheless, the findings that resistance exercise results in large increases in circulating levels of pro- and mBDNF and no change in plasma β-HB, coupled with the finding that a large increase in plasma β-HB does not affect plasma levels of mBDNF, with only a small increase in plasma pro-BDNF, suggest that β-HB is not a major regulator of muscle BDNF metabolism during exercise.

In summary, we demonstrate that pro-BDNF is clearly expressed in healthy adult human skeletal muscle, at a concentration that we quantify to approximately 40-250 pg mg^−1^ dry muscle. Pro-BDNF is also expressed in a distinct type I fiber-dependent manner, such that fiber type differences are large enough to influence whole muscle levels significantly. Both muscle and plasma levels of pro-BDNF are augmented by physical exercise in healthy humans. Exercise-mediated increases in circulating mBDNF levels likely derive, at least partly, from release of pro-BDNF from skeletal muscle, followed by cleavage to mBDNF. Elevated circulating levels of lactate and β-hydroxybutyrate during exercise are not likely to significantly enhance release of BDNF from skeletal muscle. Collectively, our findings support skeletal muscle as being a critical organ for neuroplastic signaling.

## Supplementary Material

zqae005_Supplemental_File

## Data Availability

All data are available in the main text or the supplementary materials.
