# Supplementary material for: Pro-Brain-Derived Neurotrophic Factor (BDNF), but Not Mature BDNF, Is Expressed in Human Skeletal Muscle: Implications for Exercise-Induced Neuroplasticity
Source: Function (Oxf). 2024 Jan 27;5(3):zqae005. doi: 10.1093/function/zqae005 (PMC11065112; doi:10.1093/function/zqae005)
Supplement: zqae005_Supplemental_File [file zqae005_supplemental_file.docx]

**SUPPLEMENTARY DATA FILE – Edman et al.**

**
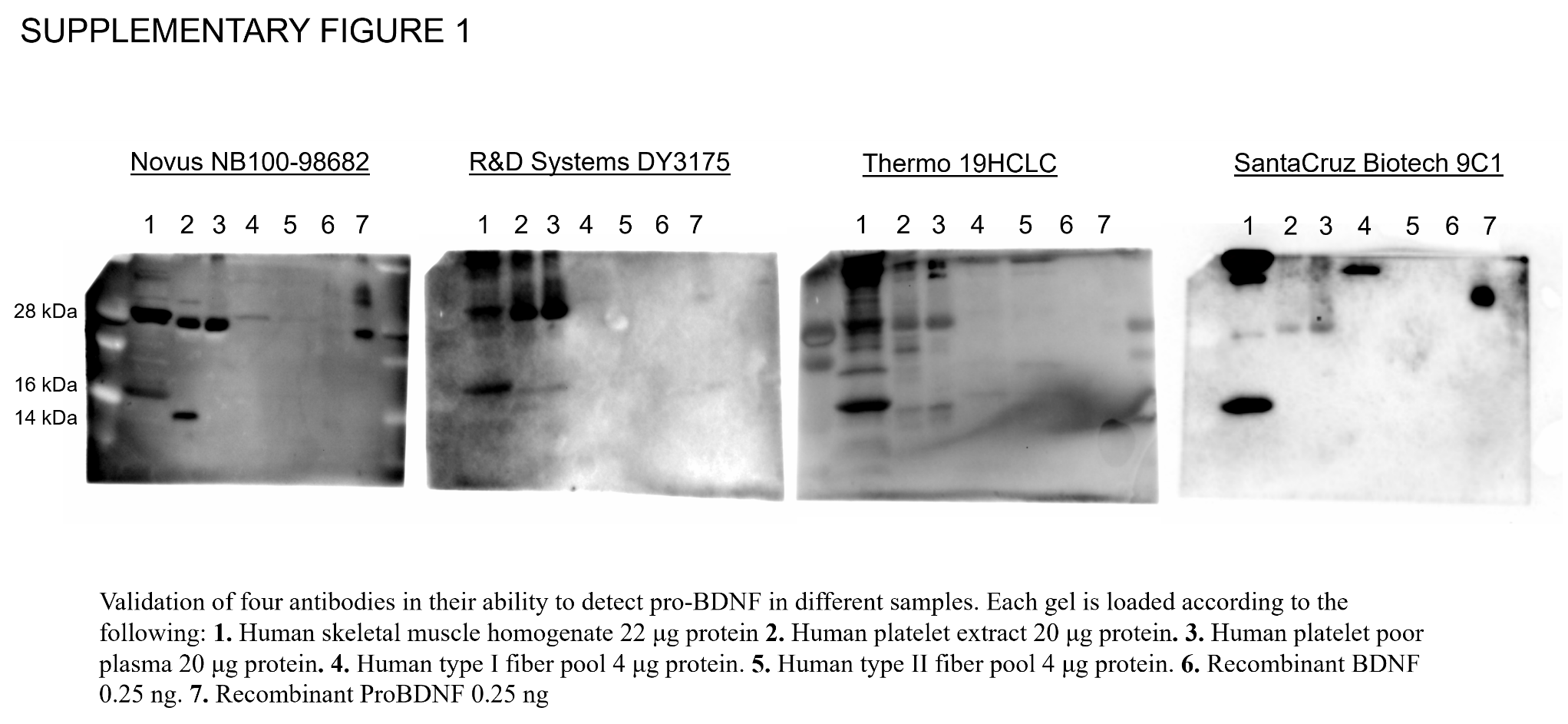
**

**STATISTICS SUPPLEMENT**

**Pro-BDNF, Muscle Fiber Pools, Immunoblotting, Fig 2A**

**Pro-BDNF, Muscle Fibers, IHC, Fig 2B**

**BDNF, Fiber type RNAseq, Fig 2C**

**Pro-BDNF, Muscle Fiber Dominance, Immunoblotting, Fig 2D and 2E**

**Pro-BDNF, Muscle Fiber Dominance, ELISA, Data not in figures.**

**Lactate Infusion Study, Plasma lactate, Fig 3A**

**Lactate Infusion Study, Plasma β-hydroxybutyrate, Fig 3B**

**Lactate Infusion Study, Plasma mBDNF, Fig 3C**

**Lactate Infusion Study, Plasma pro-BDNF, Fig 3D**

**Lactate Infusion Study, Muscle BDNF mRNA, Fig 3E**

**Lactate Infusion Study, Muscle mBDNF Immunoblotting, Data not in figure.**

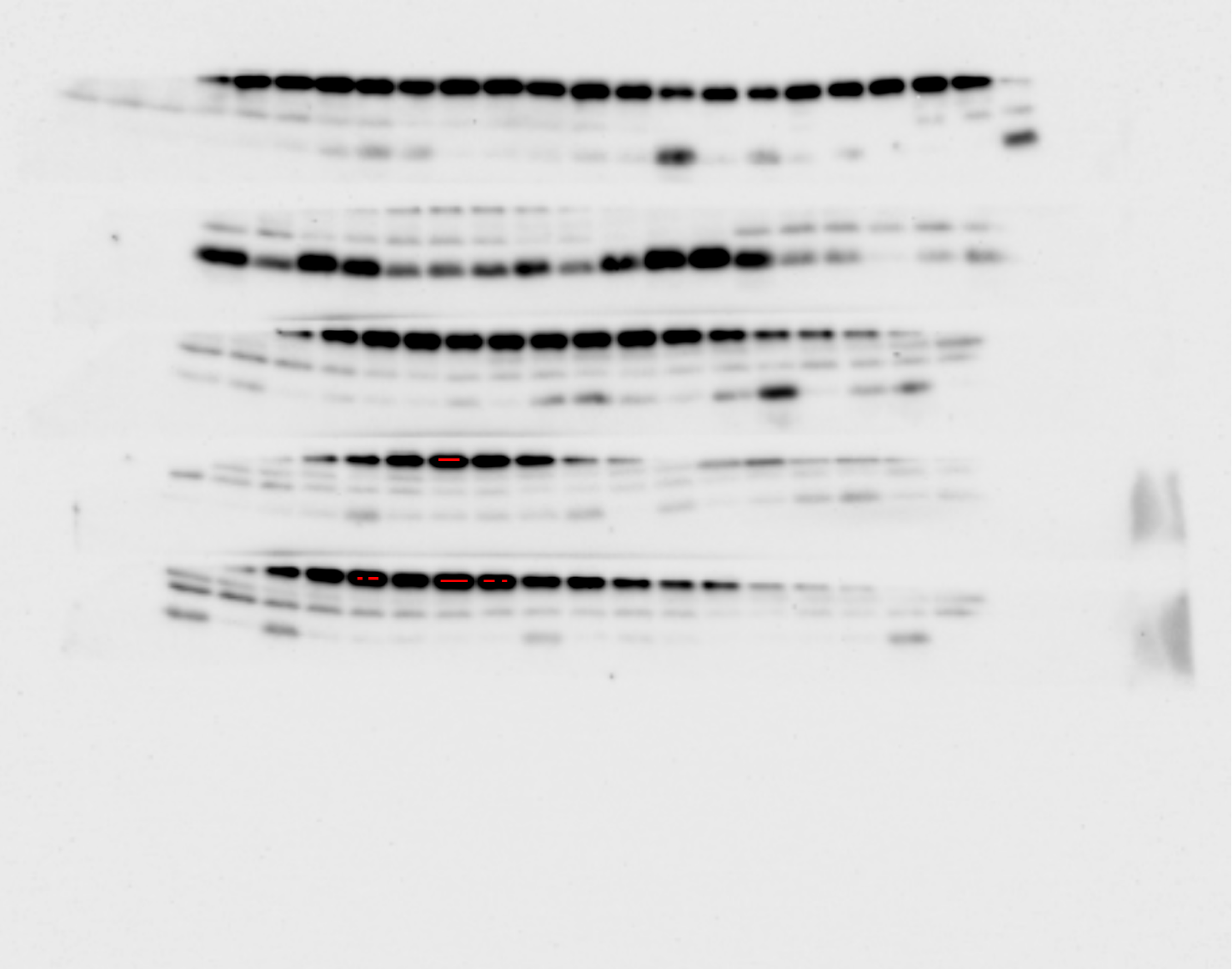


*mBDNF is the lowest band on all membrane strips. Each strip ranges from 10 - 25 kDa*

**Lactate Infusion Study, Muscle pro-BDNF Immunoblotting, Data not in figure.**

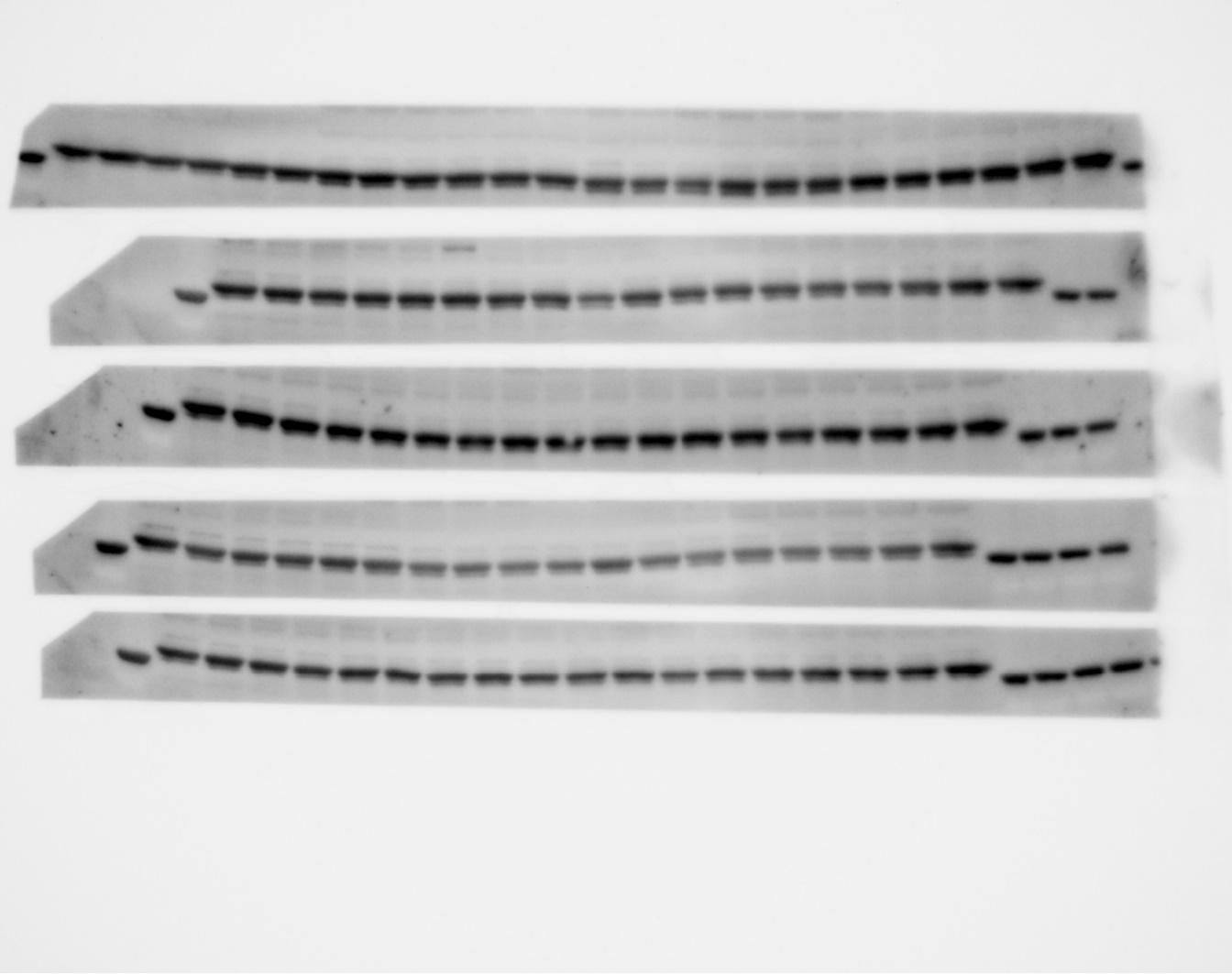


**Lactate Infusion Study, Muscle pro-BDNF ELISA, Fig 3F.**

**Fasting Study, Plasma lactate and β-hydroxybutyrate, Fig 4A and 4B.**

**Fasting Study, Plasma mBDNF and pro-BDNF, Fig 4C and 4D.**
